# Neural Advantages of Older Musicians Involve the Cerebellum: Implications for Healthy Aging Through Lifelong Musical Instrument Training

**DOI:** 10.3389/fnhum.2021.784026

**Published:** 2022-01-05

**Authors:** Masatoshi Yamashita, Chie Ohsawa, Maki Suzuki, Xia Guo, Makiko Sadakata, Yuki Otsuka, Kohei Asano, Nobuhito Abe, Kaoru Sekiyama

**Affiliations:** ^1^Graduate School of Advanced Integrated Studies in Human Survivability, Kyoto University, Kyoto, Japan; ^2^School of Music, Mukogawa Women’s University, Hyogo, Japan; ^3^Department of Behavioral Neurology and Neuropsychiatry, Osaka University United Graduate School of Child Development, Osaka, Japan; ^4^Graduate School of Social and Cultural Sciences, Kumamoto University, Kumamoto, Japan; ^5^Japan Society for the Promotion of Science, Tokyo, Japan; ^6^Institute for Logic, Language and Computation, University of Amsterdam, Amsterdam, Netherlands; ^7^Kokoro Research Center, Kyoto University, Kyoto, Japan; ^8^Faculty of Child Care and Education, Osaka University of Comprehensive Children Education, Osaka, Japan

**Keywords:** aging, older musicians, cerebellum, finger tap, music imagery

## Abstract

This study compared 30 older musicians and 30 age-matched non-musicians to investigate the association between lifelong musical instrument training and age-related cognitive decline and brain atrophy (musicians: mean age 70.8 years, musical experience 52.7 years; non-musicians: mean age 71.4 years, no or less than 3 years of musical experience). Although previous research has demonstrated that young musicians have larger gray matter volume (GMV) in the auditory-motor cortices and cerebellum than non-musicians, little is known about older musicians. Music imagery in young musicians is also known to share a neural underpinning [the supramarginal gyrus (SMG) and cerebellum] with music performance. Thus, we hypothesized that older musicians would show superiority to non-musicians in some of the abovementioned brain regions. Behavioral performance, GMV, and brain activity, including functional connectivity (FC) during melodic working memory (MWM) tasks, were evaluated in both groups. Behaviorally, musicians exhibited a much higher tapping speed than non-musicians, and tapping speed was correlated with executive function in musicians. Structural analyses revealed larger GMVs in both sides of the cerebellum of musicians, and importantly, this was maintained until very old age. Task-related FC analyses revealed that musicians possessed greater cerebellar-hippocampal FC, which was correlated with tapping speed. Furthermore, musicians showed higher activation in the SMG during MWM tasks; this was correlated with earlier commencement of instrumental training. These results indicate advantages or heightened coupling in brain regions associated with music performance and imagery in musicians. We suggest that lifelong instrumental training highly predicts the structural maintenance of the cerebellum and related cognitive maintenance in old age.

## Introduction

Normal aging accompanies the age-related decline in various cognitive and motor functions, especially in processing speed, episodic and working memory, and motor speed (Park et al., 2002; Seidler et al., 2010; Nyberg et al., 2012). Magnetic resonance imaging (MRI) studies have shown that the volumes of the cerebellum, hippocampus, basal ganglia, and frontal cortex decrease with normal aging (Raz et al., 2004, 2010; Ramanoël et al., 2018). In a progressively aging society, it is important to identify lifestyles that effectively mitigate age-related cognitive decline and brain atrophy. Playing musical instruments is a candidate for such a lifestyle because it is associated with a reduced risk of dementia (Verghese et al., 2003; Bugos et al., 2007; Hall et al., 2009). Cross-sectional behavioral studies have shown that older instrumental musicians have higher levels of non-verbal visual memory, naming, executive function, and auditory attention compared to non-musicians (Hanna-Pladdy and MacKay, 2011; Parbery-Clark et al., 2011; Hanna-Pladdy and Gajewski, 2012; Amer et al., 2013; White-Schwoch et al., 2013; Zendel and Alain, 2014; Strong and Mast, 2019). Such superiority of musicians may be accounted for by their vigorous musical training with complex physical and mental operations such as the translation of visually presented musical symbols into auditory-motor imagery, high speed and skillful execution of finger movement to realize melodies and musical impressions, and memorization of long musical phrases. However, little is known about the neural characteristics of older musicians, that is, how their brain structure and activity benefit from lifelong training and playing of a musical instrument.

Previous studies on musicians’ brains have focused on young musicians. These studies have found larger gray matter volume (GMV) in the auditory cortex, motor cortex, cerebellum, and putamen in young musicians compared to non-musicians (Gaser and Schlaug, 2003; Vaquero et al., 2016; Acer et al., 2018). Among the various brain regions where young musicians show gray matter enlargement, the cerebellum seems to be particularly relevant to behavioral functions. For example, Hutchinson et al. (2003) reported that compared to non-musicians, young keyboard players had a larger cerebellar volume, and there was a correlation between the cerebellar volume and the daily practice intensity. Furthermore, a recent study on young musicians reported that larger GMV in the cerebellum was associated with higher performance in temporal discrimination of musical tones (Paquette et al., 2017). These findings indicate that the cerebellum is critically associated with instrumental music training and musically relevant cognitive skills. As the cerebellum has been implicated in motor control (Buhusi and Meck, 2005; Stoodley and Schmahmann, 2018), accumulating daily training of musical skills may strongly influence the cerebellum. However, investigations of musicians’ brains have been limited to young participants. Do the brain regions where enlargement has been found in young musicians (the auditory cortex, motor cortex, cerebellum, and putamen) maintain the musician’s superiority into old age? As aging greatly affects the cerebellum (Raz et al., 2010; Ramanoël et al., 2018), it is possible that the musicians’ increase in cerebellar volume is not retained in old age.

In addition, there is a growing body of evidence regarding brain activation patterns in young musicians. These studies have reported that compared to non-musicians, musicians show increased activation in various cortical regions during passive listening to piano melodies (Bangert et al., 2001, 2006; Baumann et al., 2007). This increase in activation affected not only auditory-related areas but also motor-related areas such as the primary motor and premotor cortices and the cerebellum, suggesting a degree of audiomotor transformation when listening to music. In addition, musician-specific activation of the supramarginal gyrus (SMG) was observed during passive listening, suggesting the involvement of linguistic processing (Bangert et al., 2006; Baumann et al., 2007). Moreover, Notter et al. (2020) reported that the SMG is a core region of auditory perception. One notion from these findings is that the coupling between audiolinguistic and motor circuits is stronger in the brains of musicians (Bangert et al., 2006; Zatorre et al., 2007; Patel and Iversen, 2014), perhaps because of musical training and practice. In fact, a longitudinal study revealed that young non-musicians showed increased co-activation of the auditory and motor-related areas (supplementary motor area and premotor cortex) not only in instrumental training but also when listening to a learned melody after training (Wollman et al., 2018). Moreover, the notion that auditory-motor coupling is involved in music performance and listening emphasizes the use of music imagery in musicians (Lotze et al., 2003; Meister et al., 2004; Herholz et al., 2012). In general, the way in which music imagery is used by musicians can take several forms, such as mental practice away from the instrument (Johnson, 2011), silent reading of musical scores (Brodsky et al., 2008), and thinking of the ideal sound during the performance (Trusheim, 1991). Meister et al. (2004) revealed activations in the SMG and cerebellum in musicians both when they played a presented piano piece on the keyboard and when they imagined playing the presented piece. This evidence suggests a great overlap between the neural substrates involved in music imagery and music performance, and it contributes to music excellence in collaboration with daily music performance. In addition, the SMG has been implicated in phonological processing (Rauschecker, 2011), suggesting a role in converting auditory pitch information into note names. Given these multimodal properties in music, musical instrument training enhances co-activation of audiolinguistic and motor-related areas, so that the connected circuit is triggered by the auditory input alone. Interestingly, previous studies revealed that young musicians exhibited enhanced resting-state functional connectivity (FC) between the motor and multisensory cortices (e.g., auditory, visual, somatosensory, and multisensory integration regions) compared to non-musicians (Luo et al., 2012; Klein et al., 2016). However, little is known about the functional characteristics of older musicians.

Collectively, the brain structure and activity of instrumental musicians are reorganized through instrumental training over a long period. However, most studies have focused on young musicians, whereas the brain structure and activity of older musicians have received scant attention. Given the superiority in cerebellar volume in young musicians and brain atrophy, especially in the cerebellum and hippocampus, in normal aging, it is important to clarify whether lifelong involvement in playing an instrument is effective in counteracting age-related brain atrophy.

The present study addresses two research questions. First, is the superiority in brain structure and increased co-activation of audiolinguistic and motor-related areas observed in young musicians maintained in older musicians? Second, if these characteristics are retained in older musicians, does this lead to the superiority of cognitive function measured by using behavioral assessments? To answer these, we investigated brain structure and activity, including FC, during a melodic working memory (MWM) task by comparing older musicians with non-musicians. Although a passive listening task is often used to assess brain activations for music imagery, this study used the MWM task to increase the attentiveness of participants to melodic stimuli. We hypothesized that musicians would have increased activation in one or more of the regions where differences between musicians and non-musicians have been found in young populations, such as the auditory and motor cortices, cerebellum, and SMG.

## Materials and Methods

### Participants

The Psychological Research Ethics Committee of Kyoto University approved the protocol (29-P-7), and all study participants gave their informed consent. Seventy older adults (36 musicians and 34 non-musicians, aged 65–81 years) participated in this study. Musicians who had received musical instrument training for at least 20 years were recruited through a music conservatory, an amateur orchestra, and private or volunteer-supported music schools. Non-musicians with no or less than 3 years of musical experience were recruited from the Kyoto City Silver Human Resources Center, a theatrical club, and personal connections in the neighborhood. All participants were right-handed, had no cognitive impairment (Table 1; Mini-Mental State Examination [MMSE] score ≥ 27), no history of neurological, cardiovascular, or psychiatric illness, and no contraindication for MRI. Six musicians were excluded from the analysis because of claustrophobia during the MRI scan, the presence of a brain lesion, or the use of a tranquilizer. Four non-musicians were excluded because of physical deconditioning and hearing deterioration based on age-appropriate normal hearing threshold levels (35 decibels or less at frequencies of 500, 1,000, 2,000, and 4,000 Hz). In the final analysis, there were 30 instrumental musicians (19 women and 11 men) and 30 non-musicians (17 women and 13 men).

**TABLE 1:** Demographics of musicians and non-musicians.

Total (*n* = 60)	Musicians (*n* = 30)	Non-musicians (*n* = 30)	*P*-value
Sex (male/female)	11/19	13/17	0.598
Age (years)	70.8 (4.0)	71.4 (4.6)	0.595
Education (years)	15.7 (1.6)	14.9 (2.2)	0.130
Years of cognitive activity	12.7 (19.2)	18.7 (22.6)	0.274
Years of exercise	11.3 (10.6)	7.1 (10.8)	0.142
Frequency of cognitive activity	0.9 (1.6)	1.7 (2.3)	0.116
Frequency of exercise	2.9 (2.4)	2.1 (2.3)	0.154
Frequency of work	2.5 (2.1)	1.8 (1.9)	0.173
Frequency of family care	2.3 (2.9)	2.2 (3.0)	0.923
Frequency of volunteering	0.4 (1.0)	0.4 (1.0)	0.832
MMSE (score)	29.5 (0.9)	29.6 (0.6)	0.868

*Parameters are indicated as the mean (SD). P-values for age, education, cognitive activity, exercise, work, family care, volunteering, and MMSE are from t-tests for group differences. P-value for sex ratio is from Chi-square test for group differences. Frequency, days per week; MMSE, Mini-Mental State Examination; SD, standard deviation.*

The musicians had commenced musical instrument training between the ages of 3 and 16 years (Table 2; mean = 8.6 years). They had at least 22 years of experience playing a musical instrument on a regular basis (Table 2; mean = 52.7 years, range 22–70 years) and had been playing a musical instrument for more than 10 years at the time the study was conducted (Table 2; mean = 46.4 years, range 10–70 years). All musicians were actively playing an instrument at the time of this study. The instruments included piano, violin, cello, electric guitar, mandolin, wood bass, ukulele, viola, electronic clarinet, and alto saxophone. To control for the influences of other activities, the amount of time spent performing physical exercise and cognitive activities were assessed. The types of physical exercise and cognitive activities per group is elaborated in Supplementary Table 1.

**TABLE 2:** Musical instrument training status in musicians.

Instrumental musicians (*n* = 30)	Mean (*SD*)	Range
Age of commencement	8.6 (3.3)	3–16
Years of musical training	52.7 (14.6)	22–70
Years of continuous musical training	46.4 (20.2)	10–70
Current playing time (hours/day)	1.8 (1.7)	0.1–8.5

*SD, standard deviation.*

### Goldsmiths Musical Sophistication Index

To assess musical skills and behaviors in both instrumental musicians and non-musicians, we used the Japanese translated version of the Goldsmiths Musical Sophistication Index (Gold-MSI) version 1.0 (Müllensiefen et al., 2014). This 39-item scale measures six musical sophistication dimensions: active engagement (e.g., *I spend a lot of my free time doing music-related activities*), perceptual abilities (e.g., *I can compare and discuss differences between two performances or versions of the same piece of music*), emotions (e.g., *Pieces of music rarely evoke emotions for me*), singing abilities (e.g., *I only need to hear a new tune once and I can sing it hours later*), musical training (e.g., *At the peak of my interest, I practiced ___ hours per day on my primary instrument*), and general sophistication (e.g., *I enjoy writing about music, for example, on blogs and forums*). Participants provided responses to each statement on a seven-point scale (1 = *not at all* to 7 = *very*). The results showed that the musicians had higher musical abilities than the non-musicians (Table 3).

**TABLE 3:** Musical abilities according to the Gold-MSI in both groups.

Total (*n* = 60)	Musicians (*n* = 30)	Non-musicians (*n* = 30)	*P*-value
Active engagement (score)	36.9 (9.1)	20.9 (7.7)	<0.001
Perceptual abilities (score)	47.5 (7.0)	31.9 (10.2)	<0.001
Musical training (score)	34.3 (6.6)	11.6 (4.6)	<0.001
Singing ability (score)	29.2 (7.1)	16.9 (7.7)	<0.001
Emotions (score)	28.3 (5.7)	20.4 (6.2)	<0.001
General sophistication (score)	81.8 (14.7)	40.5 (15.6)	<0.001
Melody memory accuracy (%)	73.3 (13.0)	65.4 (14.9)	0.033
Memory confidence (score)	18.2 (3.1)	14.6 (3.4)	<0.001

*Data are presented as the mean (SD). P-values are from t-tests on between-group differences. Gold-MSI, Goldsmiths Musical Sophistication Index; SD, standard deviation.*

Moreover, we administered a memory test using melodies from the Gold-MSI.^1^ The melodic memory task consisted of eight-pair trials of two short melodies (containing 10–17 notes per melody), and the second melody was always presented at a different pitch level than the first one. Participants were required to judge whether the two melodies had an identical pitch interval structure and to estimate how confident they felt about their judgment on a three-point scale (1 = *I am guessing*, 2 = *I think so*, 3 = *I am totally sure*). Melodic memory accuracy was defined as the percentage of correct answers. In addition to the identity judgment for the pair of melodies, confidence rating of the judgment was obtained on a three-point scale. The results showed that the melodic memory accuracy and confidence were higher in the musicians than in the non-musicians (Table 3). Overall, musicians in this study were shown to have higher levels of musical sophistication and skills. Of note, this melodic memory task in the Gold-MSI was conducted only for behavioral performance (and confidence), and not used in brain imaging, which will be described later.

### Behavioral Measurements

Cognitive and motor functions were measured to compare musicians and non-musicians. The cognitive and motor tests consisted of many neuropsychological tests that are likely to detect differences between musicians and non-musicians (Jäncke et al., 1997; Bugos and Mostafa, 2011; Hanna-Pladdy and MacKay, 2011; Hanna-Pladdy and Gajewski, 2012; Amer et al., 2013; Fauvel et al., 2014; Strong and Mast, 2019). Cognitive tests consisted of the Japanese versions of the verbal (letter) fluency task (Lezak et al., 2012), logical memory-I and –II subtests of the Wechsler Memory Scale-Revised edition (WMS-R), visual reproduction-I and –II subtests of the WMS-R (Wechsler, 1997; Sugishita, 2000), and parts A and B of the trail making test (TMT) (Reitan and Wolfson, 1993). Motor tests consisted of finger-tapping and pegboard tasks.

The verbal fluency task was conducted to assess verbal functioning. Participants were given a word-initial sound ‘‘KA’’ and asked to generate as many words as possible in 60 s. The logical memory-I and --II subtests of the WMS-R were conducted to evaluate verbal memory. In the logical memory-I subtest, participants were asked to immediately recall two stories, one after the other. In the logical memory-II subtest, participants were asked to recall the two stories 30 min later. The visual reproduction-I and --II subtests of the WMS-R were conducted to assess non-verbal visual memory. In the visual reproduction-I subtest, participants were presented with a geometric figure on a card (A) for 10 s and asked to draw it immediately after the presentation. Another three cards (B, C, and D) were presented in the same way. In the visual reproduction-II subtest, participants were asked to draw these geometric figures on four cards 30 min later. Parts A and B of the TMT were conducted to evaluate executive function. In TMT-A, participants were asked to draw lines, sequentially connecting 25 numbers in ascending order. In TMT-B, participants were asked to draw lines alternately between numbers and letters (1, A, 2, B, etc.). The finger-tapping and pegboard tasks were conducted to evaluate functional finger dexterity. In the finger-tapping test, by using the MIDI sequencer software Domino version 1.43^2^, participants were asked to tap a computer mouse with the index finger of each hand as fast as possible for 20 s. In the pegboard task, participants were asked to turn over 20 pegs (diameter 0.5 cm × height 3.5 cm) in 20 holes carved on a square board (length 15 cm × width 18 cm × thickness 2 cm; SAKAI Medical Co., Ltd., Japan) using just one hand.

### Statistical Analysis of Behavioral Data

Behavioral data were compared between musicians and non-musicians using the unpaired two-sample *t*-test for each of the 11 measures by using IBM SPSS statistics for windows, version 25 (IBM corporation, Armonk, NY, United States).

However, multiple tests may induce type I errors, overestimating significant effects under no correction, or type II errors, underestimating significant effects under conservative correction such as that using the Bonferroni method. The resampling method (e.g., permutation) can be used to estimate adjusted *P*-values while avoiding parametric assumptions about the joint distribution of the test statistics (Dudoit et al., 2003). Here, we conducted a permutation test (Camargo et al., 2008) using MATLAB R2020a with a statistics and machine learning toolbox. For each behavioral measure, all 60 observed samples (30 musicians and 30 non-musicians) were randomized together and were resampled to obtain a dummy *t*-value. This procedure was repeated 10,000 times for each of the 11 behavioral measures. We pooled 110,000 *t*-values (10,000 resamplings × 11 behavioral measures) and created a unique permutation *t*-distribution to obtain the single adjusted α-level threshold (the top five percentile rank in the distribution).

Finally, correlation analyses (only for behavioral tests in which group differences were significant) were conducted by calculating Spearman’s rank-order correlation coefficients using IBM-SPSS. For these multiple coefficients, validation tests for correlations were performed with a permutation test using MATLAB R2020a. To examine the correlation in a given pair of variables (e.g., tapping and TMT-B), a dummy coefficient was obtained by correlating the two variables randomly across participants. This procedure was repeated 10,000 times for each of the 11 correlations. We pooled a total of 110,000 dummy coefficients (10,000 resamplings × 11 correlations) and created a unique permutation coefficient distribution to obtain the single adjusted α-level threshold (the top five percentile ranks in the distribution). For all analyses, *P* < 0.05 was considered statistically significant.

### Image Acquisition

Scanning was performed using a 3-T Siemens Magnetom Verio MR scanner (Siemens, Erlangen, Germany). The participant’s head was immobilized in a scanner head coil (12 channels). Functional images were acquired with a T2-weighted echo-planar image (EPI) (repetition time, 2,000 ms; echo time, 25 ms; flip angle, 75°; acquisition matrix, 64 × 64; field of view, 224 × 224). The EPI volume was acquired in interleaved order and consisted of 39 axial slices with a slice thickness of 3.5 mm (in-plane resolution: 3.5 mm × 3.5 mm). The EPIs were acquired during the MWM task. The first five scans in each run were discarded to allow for T1 equilibration. After the MWM task, a high-resolution structural image was acquired using an axial T1-weighted magnetization-prepared rapid gradient-echo pulse sequence (field of view: 256 × 256; matrix size: 256 × 256; voxel size: 1 mm × 1 mm × 1 mm; 208 slices).

### Functional Magnetic Resonance Imaging Tasks

A block-design functional magnetic resonance imaging (fMRI) experiment was conducted with three conditions (1-back: working memory for melodies, 0-back: hearing a melody, and rest). Melodic stimuli were presented with various three-tone equal-interval sequences of piano sounds through a pair of headphones. In the 0-back task, participants were instructed to respond when the melody had finished playing. In the 1-back task, participants were asked to judge whether the melody item was identical to the one immediately preceding it. Thus, there was no working memory required for the 0-back task, while the 1-back task required maintenance of melody information for a short time. During rest, participants were asked to relax and keep their attention on the central fixation point. Instruction and practice for the *n*-back tasks (0-back, 1-back, and rest tasks) for the fMRI experiment were given prior to the scanning, outside the scanner.

Each melody item appeared for 2,000 ms. A central fixation cross was presented throughout the inter-item interval for 2,000 ms. The task period contained twelve task blocks in total, comprising four blocks each for the 0-back and 1-back tasks, and four rest blocks; the blocks were alternated in one fMRI run. Each block lasted for 32 s, and each task block consisted of eight trials. All responses were indicated with either the index (“yes”) or middle (“no”) finger of the right hand using an MRI-compatible keypad. Participants were instructed to respond as quickly and accurately as possible.

### Image Pre-processing and Statistical Analysis of Structural Data

Voxel-based morphometry (Ashburner and Friston, 2000) was performed by using the statistical parametric mapping software SPM12 (Wellcome Department of Cognitive Neurology, London) and MATLAB R2018a (The Mathworks Inc., United States). First, the structural T1-weighted images were segmented to separate the different types of tissues: gray matter, white matter, cerebrospinal fluid, soft tissue, and skull. Thereafter, the segmented images (gray matter and white matter) were spatially normalized with DARTEL, the Diffeomorphic Anatomical Registration using Exponentiated Lie algebra (Ashburner, 2007). To preserve the absolute volume of the gray matter, modulation was performed on the normalized gray matter images by multiplying the Jacobian determinants derived from the spatial normalization. Finally, the modulated gray matter images were smoothed with an 8-mm full-width at half-maximum (FWHM) Gaussian kernel.

The individual smoothed gray matter images were entered into a second-level analysis employing a random-effects analysis within the general linear model. Previous studies have shown that brain volume is related to age, sex, education, exercise, cognitive activity, and social interaction (Erickson et al., 2011; Duan et al., 2012; Mortimer et al., 2012; Cheng, 2016; Boller et al., 2017; Firth et al., 2018; Chen et al., 2019). In order to prevent the potential influence of these factors on brain volume, the two-sample *t*-test was conducted after controlling for the effects of sex, age, educational level, and the levels of cognitive activity, exercise, work, family care, and volunteering (Table 1), as previously reported (Ramanoël et al., 2018; Wu et al., 2021). In addition, the total volume of gray matter was included as a nuisance variable to correct for global differences in gray matter, as previously reported (Vaquero et al., 2016). Moreover, an explicit mask was used to exclude noisy voxels in the statistical analysis. The statistical thresholds were set at *P* < 0.001, uncorrected for multiple comparisons at the voxel levels, and *P* < 0.05 family wise error (FWE) corrected for multiple comparisons at the cluster level. Thereafter, we identified the cluster location obtained from structural data using the anatomy toolbox in SPM12 (Eickhoff et al., 2005).

In addition, we investigated associations between age and GMV in regions of interests (ROIs). Based on the difference for structural results between groups, we extracted the ROIs of a cluster using the MarsBaR software (Brett et al., 2002). Correlation analyses were conducted using Spearman’s rank-order correlation coefficients. For these multiple coefficients, validation tests for correlation were performed using the same permutation method as described for the behavioral analyses. For correlation analyses, *P* < 0.05 was considered statistically significant.

### Image Pre-processing and Statistical Analysis of Task-Related Functional Connectivity

CONN toolbox version 18b (Whitfield-Gabrieli and Nieto-Castanon, 2012) was used to analyze task-related FC during the MWM tasks (1-back and 0-back). By using default pre-processing, the possible confounding effects of head motion artifacts, as well as cerebrospinal fluid and blood oxygen level-dependent (BOLD) signals, were defined and addressed. For denoising, signals from the white matter, cerebrospinal fluid, and motion parameters were regressed from the functional data. Data were bandpass-filtered (0.008–0.09 Hz), as previously reported (Wollman et al., 2018).

The FC analysis was conducted by using seed-to-voxel analysis. Thereafter, the seeds were set, and the ROIs were obtained from the results of the structural analysis, as previously reported (Wang et al., 2018). For the seed-to-voxel analysis, individual correlation maps were generated throughout the brain by extracting the mean task BOLD time course of each seed and calculating their correlation coefficients with the BOLD time course of each voxel. The correlation was obtained by applying the general linear model and bivariate correlation analysis weighted for the hemodynamic response function. This analysis was conducted directly in the CONN toolbox using the Fisher-transformed connectivity value. In addition to this first-level analysis for each participant, a second-level analysis was conducted for the differences in connectivity between musicians and non-musicians by using the two-sample *t*-test after controlling for the effects of sex, age, educational level, and the levels of cognitive activity, exercise, work, family care and volunteering (Table 1). The statistical thresholds were set at *P* < 0.001 uncorrected for multiple comparisons at the voxel levels, and *P* < 0.05 FWE corrected for multiple comparisons at the cluster level. Finally, correlation analyses were conducted between the FC value and behavioral measures using IBM-SPSS after controlling for the effects of age, educational level, and the levels of cognitive activity and exercise. For these multiple correlation coefficients, validation tests for correlation were performed by using the same permutation method as described for the behavioral analyses. For correlation analyses, *P* < 0.05 was considered statistically significant.

### Image Pre-processing and Statistical Analysis of Functional Data

The fMRI data pre-processing was carried out using SPM12 and MATLAB R2018a. First, the origin of the EPI was manually set to the anterior–posterior commissure for each participant. The fMRI data were spatially realigned to the first volume of the series and subsequently to the across-run mean volume, after which they were co-registered with the anatomical data. The anatomical data were normalized to the Montreal Neurological Institute (MNI) space using a unified segmentation procedure. Afterward, the resulting deformation parameters were applied to the fMRI data that were resampled into 3 mm × 3 mm × 3 mm voxels and smoothed with an 8-mm FWHM Gaussian kernel.

Activated voxels during MWM activation were identified using a statistical model containing a boxcar function convolved with a canonical hemodynamic response function. A high-pass filter (1/128 Hz) was used to remove low-frequency noise, and an autoregressive (AR (1)) model was employed to correct temporal autocorrelations. The data were analyzed using the two-way ANOVA with groups (musicians and non-musicians) and tasks (0-back, 1-back, and rest tasks) after controlling for the effects of sex, age, educational level, and the levels of cognitive activity, exercise, work, family care and volunteering (Table 1), as previously reported (Huang et al., 2020). The statistical thresholds were set at *P* < 0.001 uncorrected for multiple comparisons at the voxel level, and *P* < 0.05 FWE corrected for multiple comparisons at the cluster level. Afterward, we identified the cluster location as described for the structural data analysis.

In addition, we investigated associations between musical traits and task-related parameter estimates of BOLD values in ROIs. Based on the differences between groups for functional results, we extracted the ROIs of a cluster using the MarsBaR software. Significance in Spearman’s rank-order correlation coefficients was assumed at *P* < 0.05 in IBM-SPSS. For these multiple correlation coefficients, validation tests for correlation were performed using the same permutation method as described for the behavioral analyses.

## Results

### Demographics

Demographic data are shown in Table 1. There were no significant group differences in age (musicians: 70.8 ± 4.0 years; non-musicians: 71.4 ± 4.6 years), years of education (musicians: 15.7 ± 1.6; non-musicians: 14.9 ± 2.2), MMSE (musicians: 29.5 ± 0.9; non-musicians: 29.6 ± 0.6), sex ratio, or time spent on cognitive activity, exercise, work, family care, and volunteering. These results indicate that the two groups did not significantly differ demographically.

### Behavioral Scores

**FIGURE 1:**
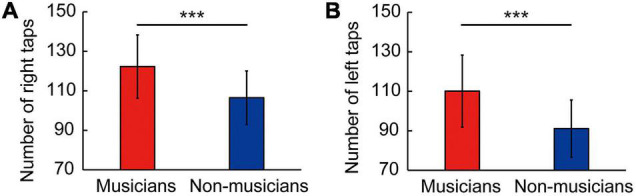
Skillful tapping in musicians. Compared to non-musicians, musicians showed enhanced performances in right tapping speed **(A)** and left tapping speed **(B)**. Parameters are indicated as the mean (*SD*). ***P < 0.001 vs. non-musicians. *SD*, standard deviation.

Behavioral data are shown in Figure 1 and Table 4 as the mean ± standard deviation (*SD*). Student’s *t*-test showed significant differences between groups in right tapping (Figure 1A: *t*_(58)_ = 4.11, *P* < 0.001, *d* = 1.06), left tapping (Figure 1B: *t*_(58)_ = 4.46, *P* < 0.001, *d* = 1.15), and verbal fluency (Table 4: *t*_(58)_ = 2.14, *P* = 0.036, *d* = 0.55). For TMT-B, Welch’s *t*-test was used due to heteroscedasticity, and the results showed a significant difference between the groups (Table 4: *t*_(44.94)_ = 2.33, *P* = 0.024, *d* = 0.60). These *t*-values were higher than the adjusted significance level threshold *t*_(58)_ = 1.99 obtained in the permutation test. In contrast, there were no significant between-group differences in the logical memory-I and –II subtests, visual reproduction-I and –II subtests, TMT-A, and pegboard task (Table 4). These results indicate that musicians had higher levels of verbal functioning, executive control, and tapping speed compared to non-musicians.

**TABLE 4:** Behavioral results in both groups.

Total (*n* = 60)	Musicians (*n* = 30)	Non-musicians (*n* = 30)	*P*-value
Verbal fluency (words)	14.1 (4.0)	12.1 (3.5)	0.036
TMT-A (second)	32.6 (10.5)	38.3 (12.2)	0.059
TMT-B (second)	70.1 (17.9)	85.9 (32.7)	0.024
Logical memory-I (score)	23.9 (6.7)	25.7 (7.4)	0.339
Logical memory-II (score)	20.9 (7.7)	21.4 (8.6)	0.789
Visual reproduction-I (score)	35.2 (5.0)	35.6 (5.6)	0.771
Visual reproduction-II (score)	34.4 (6.1)	32.1 (8.7)	0.235
Right pegboard (second)	37.1 (10.5)	39.1 (6.3)	0.375
Left pegboard (second)	39.9 (9.3)	42.0 (7.1)	0.331

*Data are presented as the mean (SD). P-values are from t-tests on group differences. TMT, trail making test; Logical memory-I, immediate recall; Logical memory-II, delayed recall; Visual reproduction-I, immediate recall; Visual reproduction-II, delayed recall; SD, standard deviation.*

**FIGURE 2:**
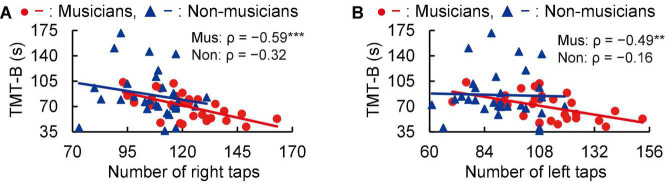
Correlation between tapping and TMT-B. In musicians, the numbers of right **(A)** and left **(B)** tapping (in 20 s) were negatively correlated with the time to complete the TMT-B. By contrast, these correlations with tapping were not statistically significant in non-musicians. **P < 0.01, ***P < 0.001, Mus, musicians; Non, non-musicians; TMT, trail making test.

In subsequent correlation analyses, tapping scores were negatively correlated with time to perform the TMT-B in musicians (right: ρ = −0.59, *P* < 0.001; left: ρ = −0.49, *P* = 0.006; Figure 2). These Spearman’s ρ-values were higher in absolute value than the adjusted significance level threshold |ρ| = 0.36 obtained in the permutation test. By contrast, non-musicians did not show such a correlation (right: ρ = −0.32, *P* = 0.082; left: ρ = −0.16, *P* = 0.399; Figure 2). These results indicate that for the musicians, higher tapping speeds were linked to more rapid executive control.

### Musical Instrument Experience-Related Structural Changes

**FIGURE 3:**
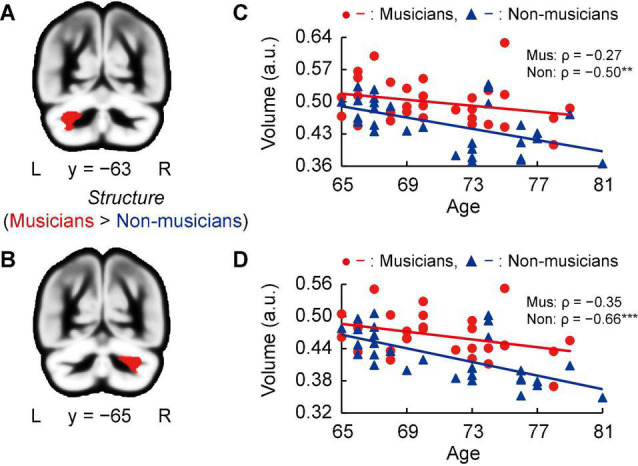
Structural changes related to musical instrument experience. **(A,B)** Musicians had larger GMVs in both sides of the cerebellum compared to non-musicians. **(C,D)** For the cerebellar ROIs in both hemispheres, the non-musicians displayed negative correlations between their GMV and age. In musicians, such correlations (age-related volume reductions) were not significant. **P < 0.01, ***P < 0.001, Mus, musicians; Non, non-musicians; GMV, gray matter volume; ROI, region of interest; L, left; R, right; a.u., arbitrary units.

Whole-brain voxel-based gray matter analyses in both groups showed significantly larger GMVs in crus I of the left and right cerebellum of musicians compared to those of non-musicians (Figures 3A,B, and Supplementary Table 2). Subsequently, we investigated associations between the GMV of ROIs in bilateral crus I of both sides of the cerebellum and age (Figures 3C,D). In non-musicians, the GMVs of crus I of both sides of the cerebellum were negatively correlated with age (left: ρ = −0.50, *P* = 0.005; right: ρ = −0.66, *P* < 0.001), indicating that in non-musicians, these bilateral regions of the cerebellum are more subject to age-dependent atrophy. These Spearman’s ρ-values were higher in absolute value than the adjusted significance level threshold |ρ| = 0.36 obtained in the permutation test. By contrast, such correlations with age were not significant in musicians (left: ρ = −0.27, *P* = 0.147; right: ρ = −0.35, *P* = 0.058). These results show that crus I of both sides of the cerebellum exhibited differential age-related GMV changes between musicians and non-musicians.

### Task-Related Functional Connectivity

**FIGURE 4:**
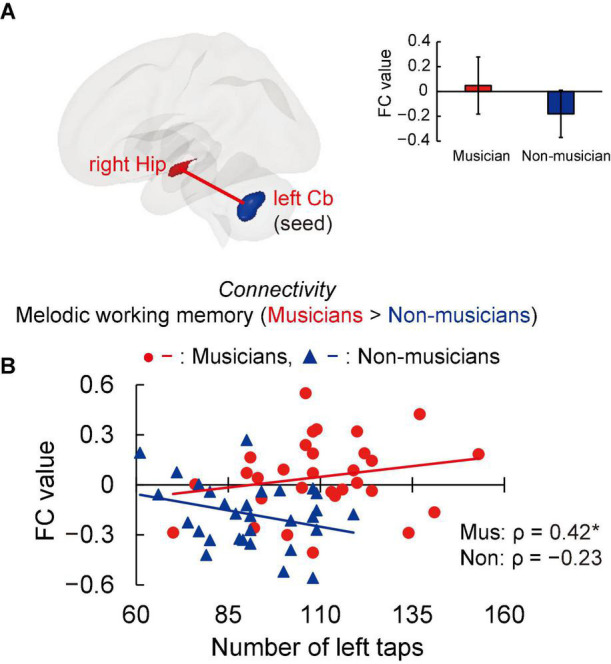
Task-related FC. **(A)** Using ROIs in both sides of the cerebellum as the seed (where musicians showed greater GMV than non-musicians, as illustrated in Figure 3), musicians exhibited enhanced FC between the left cerebellum and right hippocampus during the MWM task compared to non-musicians, but no change in FC of the right cerebellum and other regions. **(B)** In the associations between cerebellar-hippocampal FC and behavioral functions, task-related cerebellar-hippocampal FC and left tapping speed were positively correlated in musicians. *P < 0.05, Mus, musicians; Non, non-musicians; FC, functional connectivity; ROI, region of interest; GMV, gray matter volume; MWM, melodic working memory; Hip, hippocampus; Cb, cerebellum.

Structural analyses revealed larger GMVs in both sides of the cerebellum in musicians compared to non-musicians. Therefore, we used ROIs in both sides of the cerebellum as the seed for the FC analyses of the task-related fMRI data. The group differences in FC are shown in Figure 4A and listed in Supplementary Table 3. Compared to non-musicians, musicians had enhanced FC between the left cerebellum and the right hippocampus. In addition, we investigated the correlation between cerebellar-hippocampal FC and behavioral functions (Figure 4B). The task-related cerebellar-hippocampal FC and left tapping speed were significantly correlated in musicians (ρ = 0.42, *P* = 0.039). This Spearman’s ρ was higher in absolute value than the adjusted significance level threshold |ρ| = 0.36 obtained in the permutation test. By contrast, non-musicians did not show such a correlation (ρ = −0.23, *P* = 0.284). These results indicate that in musicians, enhancement of the cerebellar-hippocampal FC in MWM processing is linked to a higher tapping speed.

### Task-Related Activation

**FIGURE 5:**
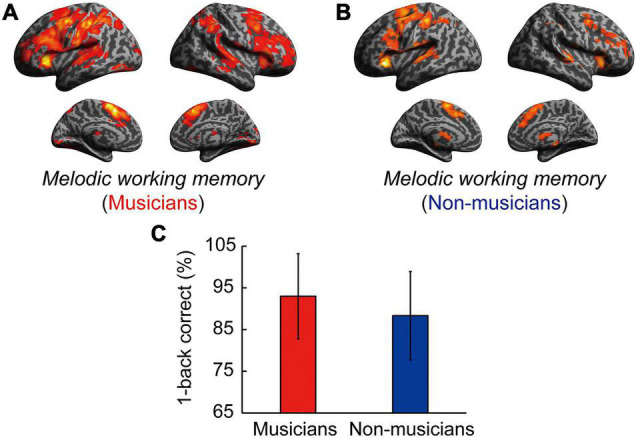
Task-related brain activity in each group. **(A)** Musicians have increased activation of extensive brain regions. **(B)** Non-musicians have increased activation of limited brain regions. **(C)** There were no significant between-group differences in 1-back task performance. For significant between-group differences, see Supplementary Table 4, Figure 6, and the main text.

**FIGURE 6:**
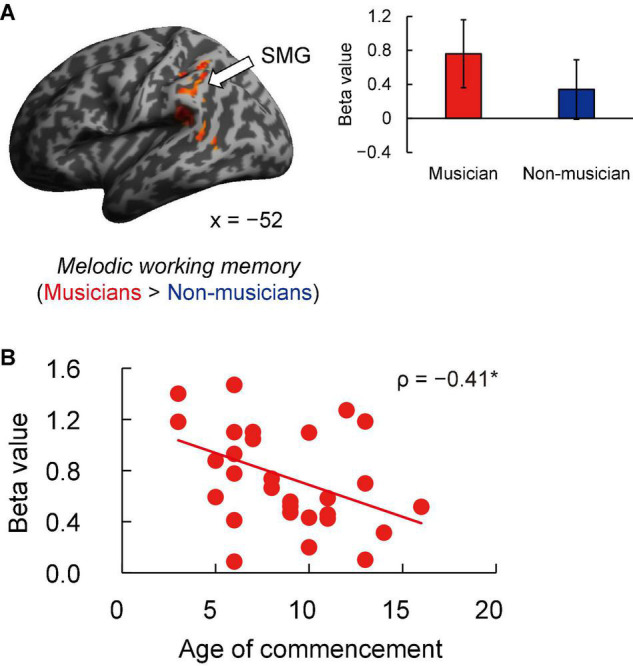
Task-related brain activity. **(A)** Musicians exhibited increased activation of the left SMG during the MWM task (1-back vs. rest), whereas no change was seen in auditory and motor regions. **(B)** Using ROIs in the left SMG, age at commencement of musical training was negatively correlated with task-related SMG activation in musicians. *P < 0.05, SMG, supramarginal gyrus; MWM, melodic working memory; ROI, region of interest.

Contrast images for 1-back vs. rest were computed to assess the brain activity during the MWM task in both groups. Non-musicians had increased activation in limited brain regions, whereas musicians had increased activation in extensive brain regions (Figures 5A,B). In terms of behavioral performance during the MWM task, there were no significant between-group differences in 1-back task performance (Figure 5C). The other between-group differences are shown in Figure 6A and listed in Supplementary Table 4. Musicians showed greater activation within the left SMG than non-musicians. However, contrast images for 0-back vs. rest and 1-back vs. 0-back did not show a significant difference between groups. Subsequently, we extracted parameter estimates in the ROI of the left SMG in musicians and investigated the associations between its activation and musical traits (Figure 6B). No correlation was found between SMG activation and scores of behavioral performances. One trait, i.e., the age at the commencement of musical training, was negatively correlated with SMG activation (ρ = −0.41, *P* = 0.026). This Spearman’s ρ was higher in absolute value than the adjusted significance level threshold |ρ| = 0.36 obtained in the permutation test. The activation during the MWM task was higher for the musicians with earlier training commencement, indicating an influence of childhood instrumental training.

## Discussion

The present work aimed to investigate the association between lifelong instrumental training and age-related cognitive decline and brain atrophy. The main findings revealed that compared to non-musicians, musicians (i) showed enhanced performance in several constructs such as tapping speed (Figure 1), verbal fluency, and executive control (Table 4); (ii) had larger GMVs in both sides of the cerebellum (Figure 3); (iii) exhibited higher task-related FC between the left cerebellum and right hippocampus, with FC being correlated with tapping speed (Figure 4); and (iv) exhibited increased activation of the left SMG during the MWM task, with its greater activation being associated with earlier commencement of musical training (Figure 6).

### The Effect of Lifelong Musical Training: From Skillful Tapping to Cognition

The behavioral results revealed that musicians had higher verbal fluency, TMT-B, and finger-tapping performances compared to non-musicians. Previous studies have reported that young musicians have higher levels of tapping speed (Jäncke et al., 1997), finger tap-related rhythm synchronization (Karpati et al., 2016), and executive function (Bugos and Mostafa, 2011) than young non-musicians. Further studies indicated older musicians’ superiority in letter fluency and executive function in cognitive aging (Hanna-Pladdy and MacKay, 2011; Hanna-Pladdy and Gajewski, 2012; Amer et al., 2013; Fauvel et al., 2014; Strong and Mast, 2019; Chan and Alain, 2020), which our results support. Moreover, we provided a correlation between tapping speed of simple finger movement and executive function measured by using the TMT-B in older musicians, in line with a previous study that displayed simple finger taps and executive control association for non-musicians (Mak et al., 2016). In contrast, some studies have reported that complex finger taps in non-musicians had a high load in executive function compared to simple finger taps (Harding et al., 2017; Holm et al., 2017). Because these studies showed within-non-musicians differences in levels of load of executive function for different complexity of tapping, the relationship between the previous findings and our between-groups difference cannot be clearly interpreted. Since musical performance requires motor planning and monitoring of finger movement (Palmer and Drake, 1997; Moreno et al., 2011; Shen et al., 2019; Colombo et al., 2020; Guo et al., 2021), we suggest that musicians perform finger taps with higher executive involvement compared to non-musicians even when they are simple ones. The higher levels of executive function in musicians can be maintained by the same processes to enable masterful temporal and ordinal precision of finger movements, together with fine-grained finger force control. Although aging degrades processing speed and working memory associated with executive function (Park et al., 2002; Nyberg et al., 2012), the preservation of skillful tapping abilities through instrumental training may mitigate such declines in older adults.

In addition, the musicians in our study displayed behavioral superiority in verbal (letter) fluency, in line with previous studies that addressed music and language associations (Anvari et al., 2002; Patel and Iversen, 2007; Wong et al., 2007; Kraus and Chandrasekaran, 2010). Letter fluency has been shown to involve phonological processing (Heim et al., 2008; Shao et al., 2014). Patel and Iversen (2007) reported that musical training strengthens the processing of auditory features common to music and language. Based on these associations, musicians’ superior verbal fluency in our study may be explained by the common phonological features in music-making and language production (Patel and Iversen, 2007; Wong et al., 2007). As learning about the acoustic structure of musical pieces involves phonological processing, music-making may facilitate the higher performance of letter fluency in musicians compared to non-musicians. Another study emphasized that music training involves a high working memory load, maintenance of selective attention skills, and learning of the acoustic rules that bind musical sounds together (Kraus and Chandrasekaran, 2010). These cognitive skills are also crucial for speech production (Strait et al., 2011); such an overlap may partly explain this intriguing positive transfer effect from music to language.

### Older Musicians’ Cerebellar Maintenance

Whole-brain voxel-based GMV analyses revealed significantly larger bilateral GMVs in crus I of the cerebellum in musicians compared to non-musicians. Moreover, we found that the two groups showed different age-related volume changes in cerebellar ROIs; whereas non-musicians’ cerebellar GMVs in the ROIs sharply decreased with age, musicians did not show a significant correlation between volume and age. These findings indicate that musical instrument training possibly induces neural structural advantages in two key ways. First, consistent with previous findings of a larger cerebellar GMVs in young musicians compared to non-musicians (Gaser and Schlaug, 2003; Acer et al., 2018), we demonstrated the superiority of older musicians compared to age-matched non-musicians. Second, the cerebellar volume is known to decrease with age (Raz et al., 2010; Ramanoël et al., 2018), and it is important to verify whether lifelong engagement in playing a musical instrument suppresses this reduction. We found that musicians may maintain their cerebellar volume into old age, whereas the cerebellum of a non-musician is relatively vulnerable to age-related atrophy. These results suggest that the lifelong practice of a musical instrument is associated with structural maintenance of cerebellum in old age.

The cerebellum is known to be involved in some cognitive control processes and play an important role in fine motor activity (Buhusi and Meck, 2005; Hautzel et al., 2009; Stoodley and Schmahmann, 2018). In addition, several studies have proposed that there is a functional topographic organization in the cerebellum such that sensorimotor function is represented predominantly in the anterior lobe (lobules I–V), cognitive processing is associated with posterior lobules VI and VII (including crus I, II, and VIIB), and the cerebellar vermis and fastigial nuclei constitute the limbic cerebellum (Schmahmann, 2004, 2019; Stoodley and Schmahmann, 2009). The cluster of cerebellar GMV that showed between-group differences in our study was located in crus I according to the coordinate system of Eickhoff et al. (2005). Some studies have reported that reduction in GMV of crus I is associated with executive dysfunction (Olivito et al., 2018; Tse et al., 2020), suggesting that such structural changes play an important role in the maintenance of motor planning and monitoring (D’Agata et al., 2011; Stoodley, 2012; Stoodley and Schmahmann, 2018), which are needed for skillful execution of finger movement during music-making. This region also corresponds to an area of musically relevant temporal discrimination in the cerebellum (Baer et al., 2015; Paquette et al., 2017). For efficient predictive adaptation of behavior, the cerebellum is thought to play key roles in the precise encoding of perceived temporal structures (Schwartze and Kotz, 2013). Several studies have pointed out that the cerebellum is involved in optimizing sensory input from the auditory system when stimulated by music (Gao et al., 1996; Penhune et al., 1998). These aspects of musical training could contribute to the structural superiority in crus I of the cerebellum in old age. In particular, crus I enlargement may help to coordinate auditory and motor systems in musicians.

Interestingly, although increased GMVs of the auditory and motor cortices has been reported in young professional musicians (Gaser and Schlaug, 2003; Groussard et al., 2010; Vaquero et al., 2016; Acer et al., 2018), the present study did not find such between-group differences in these regions in older participants. This inconsistency may be explained by differences in the expertise level of musicians (e.g., professional vs. amateur). Of note, our results, based on an uncorrected threshold (*P* < 0.001), showed that older musicians had a larger GMV in the right hippocampus compared to non-musicians (Supplementary Figure 1A and Supplementary Table 5). Moreover, the hippocampal GMV in the ROIs decreased with age in older participants, including both musicians and non-musicians (Supplementary Figure 1B). Although the uncorrected results should be interpreted with caution, these results suggest the possibility that musical instrument training suppresses the reduction in hippocampal volume. Such suppression may be explained by the role of the hippocampus in temporal processing and consonance/dissonance detection (Wieser and Mazzola, 1986; James et al., 2008; Groussard et al., 2010). The regular and sustained practice of musical instruments may affect the cerebellum and hippocampus in old age more strongly than the auditory and motor cortices, presumably because their function in temporal processing is essential for playing an instrument.

### Activation of the Cerebellar-Hippocampal Network and Supramarginal Gyrus in Musicians

We found that musicians had enhanced FC between the left cerebellum and right hippocampus in the MWM task compared to non-musicians. Moreover, higher cerebellar-hippocampal FC was associated with greater left tapping speed in musicians, indicating a musician-specific link between processes controlling finger movements and cerebellar-hippocampal FC during the MWM task. This result suggests that musicians try to maintain their MWM by associating a melody with imaginary finger movements.

A few fMRI studies have reported that activation of the cerebellum was observed in imagery tasks involving specific timing and sequential finger coordination, such as imagery of playing the piano and a finger-tapping task (Hanakawa et al., 2003; Meister et al., 2004), suggesting a role of the cerebellum in the imagery of music-related finger movements. Anatomically, the fiber bundles of the posterior cerebellum (e.g., crus I) project to the hippocampus where they play a role in motor control and cognitive function (Arrigo et al., 2014; Bohne et al., 2019). The heightened cerebellar-hippocampal FC and its association with tapping speed in musicians in our study suggest that during MWM tasks, musicians may encode melodies to sequences of finger movements for memory maintenance. Alternatively, the heightened cerebellar-hippocampal FC may be related to music imagery. Alonso et al. (2016) reported that non-musicians had increased activation in the left cerebellum and right hippocampus in a music imagery task in which participants were instructed to bind lyrics and melodies together to encode a song. This finding supports the involvement of the cerebellar-hippocampal FC in music imagery. Thus, it is natural to anticipate that such a music imagery-related network would be strengthened in musicians through musical instrument training.

Another feature of musicians’ fMRI data was increased activation of the left SMG in the MWM task compared to non-musicians. Moreover, in musicians, greater activation of the left SMG was associated with earlier commencement of instrumental training. That is, learning a musical instrument from childhood is closely linked to increased activation of the left SMG in MWM processes.

In fMRI studies of music imagery and performance (using fingers), activation of the SMG and cerebellum has been observed in musicians during both imagery and performance conditions (Lotze et al., 2003; Meister et al., 2004; Herholz et al., 2012), indicating that music imagery shares the same neural substrates in these regions with executed music performance. The increased SMG activation in our musicians suggests that musicians evoked music imagery during the MWM task. The SMG has also been implicated in auditory perception (Rauschecker, 2011; Notter et al., 2020), such as phonological processing. A previous study reported that in young developing children aged 5–6 years, specialization of the left SMG in phonological processing is already evident (Weiss et al., 2018). In addition, several studies have shown that activation of the left SMG is associated with the phonological short-term memory task (Paulesu et al., 1993; Gaab et al., 2003, 2006; Lerub et al., 2021), suggesting that the left SMG is the primary location of the phonological store. Furthermore, SMG activation was observed during both linguistic auditory imagery and listening to novel piano melodies (Hickok et al., 2003; Tsai et al., 2018). In our study, we think that musicians may have encoded auditory pitch information into linguistic labels, such as note names (“C,” “D,” and “E”) during the MWM task for working memory maintenance, more so than non-musicians. This interpretation is also consistent with the correlation between SMG activation and earlier commencement of instrumental training.

Although increased activation of the auditory and motor cortices during melody-listening has been reported in young musicians (Bangert et al., 2001, 2006; Baumann et al., 2007), we did not find such between-group differences in these regions during the MWM task in the older participants of the present study. One possible reason is the difference in stimuli: stimuli in our MWM task were not long phrases from existing musical pieces as in previous studies, but short sequences of three tones in a uniform rhythm, which may have evoked less musical processing. Another possibility is the difference in tasks: the previous studies used a passive listening task, but we used a working memory task.

Taken together, cerebellar-hippocampal FC and the SMG seem to play a role in encoding the melodies to motor (finger movements) and phonetic (note names) information, respectively. This notion indicates musicians’ advantages or heightened coupling in brain regions associated with music performance and imagery.

Our study has some limitations. First, our study was cross-sectional; thus, the superiority of musicians observed in several aspects cannot be attributed to musical instrument training in a causal manner. However, our careful control of various confounding factors together with observed correlations between musical traits and cognitive or neural characteristics increase the likelihood that the results support the hypothesis that lifelong musical training is useful in maintaining cognitive function in old age. Second, the musicians in our study started their musical training in relatively early periods, by the age of 16 years at the latest. Therefore, the superiority of brain functions in older individuals who started their musical instrument training beyond the age of 16 remains unknown. Third, the present study was unable to recruit a sufficient number of musicians for each instrument to investigate the effects of musical instrument type on the brain and cognitive functions. Therefore, it is unclear how the musical instrument type influenced the results in this study. However, the participation of a heterogenous group of musicians in our study may improve the generalizability of our results relative to those of previous studies (Gaser and Schlaug, 2003; Hutchinson et al., 2003; Bangert et al., 2006; Acer et al., 2018). Furthermore, as a musicians age, they may display training-related mitigation of subcortical atrophy in additional regions such as the hippocampus. To investigate this hypothesis, longitudinal studies of the brain structure of older musicians are needed.

## Conclusion

For the first time, we found neural advantages in older musicians compared to age-matched non-musicians. The musicians showed lower levels of age-related cognitive decline and brain atrophy in the cerebellum. Behaviorally, cerebellum-related skillful tapping was associated with the maintenance of executive function in musicians. Moreover, fMRI results during the MWM task indicated musician-specific heightened cerebellar-hippocampal FC and SMG activation, suggesting that musicians may encode melodies into motor and phonetic information for memory maintenance. The present results demonstrate that lifelong active engagement in musical instrument training is related to structural and functional advantages in the neural system involving the cerebellum. The main findings of the present study shed new light on how lifelong musical instrument training potentially influences brain maintenance in old age.

## Data Availability Statement

The raw data supporting the conclusions of this article will be made available by the authors, without undue reservation.

## Ethics Statement

The studies involving human participants were reviewed and approved by the Psychological Research Ethics Committee of Kyoto University, (protocol 29-P-7). The patients/participants provided their written informed consent to participate in this study.

## Author Contributions

KS and CO designed the research. MY, CO, and XG performed the research. MY analyzed the data. MY and KS wrote the manuscript. All authors contributed to the experimental materials and tools, and revised the manuscript.

## Conflict of Interest

The authors declare that the research was conducted in the absence of any commercial or financial relationships that could be construed as a potential conflict of interest.

## Publisher’s Note

All claims expressed in this article are solely those of the authors and do not necessarily represent those of their affiliated organizations, or those of the publisher, the editors and the reviewers. Any product that may be evaluated in this article, or claim that may be made by its manufacturer, is not guaranteed or endorsed by the publisher.

## Footnotes

1
https://www.gold.ac.uk/music-mind-brain/gold-msi/download/

2
http://takabosoft.com/domino

## References

[B1] Acer N. Bastepe-Gray S. Sagiroglu A. Gumus K. Z. Degirmencioglu L. Zararsiz G. (2018). Diffusion tensor and volumetric magnetic resonance imaging findings in the brains of professional musicians. *J. Chem. Neuroanat.* 88 33–40. 10.1016/j.jchemneu.2017.11.003 29113947

[B2] Alonso I. Davachi L. Valabregue R. Lambrecq V. Dupont S. Samson S. (2016). Neural correlates of binding lyrics and melodies for the encoding of new songs. *Neuroimage* 127 333–345. 10.1016/j.neuroimage.2015.12.018 26706449

[B3] Amer T. Kalender B. Hasher L. Trehub S. E. Wong Y. (2013). Do older professional musicians have cognitive advantages? *PLoS One* 8:e71630. 10.1371/journal.pone.0071630 23940774 PMC3737101

[B4] Anvari S. H. Trainor L. J. Woodside J. Levy B. A. (2002). Relations among musical skills, phonological processing, and early reading ability in preschool children. *J. Exp. Child Psychol.* 83 111–130. 10.1016/s0022-0965(02)00124-8 12408958

[B5] Arrigo A. Mormina E. Anastasi G. P. Gaeta M. Calamuneri A. Quartarone A. (2014). Constrained spherical deconvolution analysis of the limbic network in human, with emphasis on a direct cerebello-limbic pathway. *Front. Hum. Neurosci.* 8:987. 10.3389/fnhum.2014.00987 25538606 PMC4259125

[B6] Ashburner J. (2007). A fast diffeomorphic image registration algorithm. *Neuroimage* 38 95–113. 10.1016/j.neuroimage.2007.07.007 17761438

[B7] Ashburner J. Friston K. J. (2000). Voxel-based morphometry–the methods. *Neuroimage* 11 805–821. 10.1006/nimg.2000.0582 10860804

[B8] Baer L. H. Park M. T. Bailey J. A. Chakravarty M. M. Li K. Z. Penhune V. B. (2015). Regional cerebellar volumes are related to early musical training and finger tapping performance. *Neuroimage* 109 130–139. 10.1016/j.neuroimage.2014.12.076 25583606

[B9] Bangert M. Haeusler U. Altenmüller E. (2001). On practice: how the brain connects piano keys and piano sounds. *Ann. N.Y. Acad. Sci.* 930 425–428. 10.1111/j.1749-6632.2001.tb05760.x 11458857

[B10] Bangert M. Peschel T. Schlaug G. Rotte M. Drescher D. Hinrichs H. (2006). Shared network for auditory and motor processing in professional pianists: evidence from fMRI conjunction. *Neuroimage* 30 917–926. 10.1016/j.neuroimage.2005.10.044 16380270

[B11] Baumann S. Koeneke S. Schmidt C. F. Meyer M. Lutz K. Jancke L. (2007). A network for audio-motor coordination in skilled pianists and non-musicians. *Brain Res.* 1161 65–78. 10.1016/j.brainres.2007.05.045 17603027

[B12] Bohne P. Schwarz M. K. Herlitze S. Mark M. D. (2019). A new projection from the deep cerebellar nuclei to the hippocampus via the ventrolateral and laterodorsal thalamus in mice. *Front. Neural. Circuits* 13:51. 10.3389/fncir.2019.00051 31447652 PMC6695568

[B13] Boller B. Mellah S. Ducharme-Laiberte G. Bellevillé S. (2017). Relationships between years of education, regional grey matter volumes, and working memory-related brain activity in healthy older adults. *Brain Imaging Behav.* 11 304–317. 10.1007/s11682-016-9621-7 27734304

[B14] Brett M. Anton J.-L. Valabregue R. Poline J.-B. (2002). “Region of Interest Analysis Using an SPM Toolbox,” in *[Abstract] Presented at the 8th International Conference on Functional Mapping of the Human Brain, June 2-6, 2002, Sendai, Japan. Retrieved from CD-ROM in Neuroimage 16*, (Sendai).

[B15] Brodsky W. Kessler Y. Rubinstein B. S. Ginsborg J. Henik A. (2008). The mental representation of music notation: notational audiation. *J. Exp. Psychol. Hum. Percept. Perform.* 34 427–445. 10.1037/0096-1523.34.2.427 18377180

[B16] Bugos J. Mostafa W. (2011). Musical training enhances information processing speed. *Bull. Council. Res. Music Edu.* 187 7–18. 10.2307/41162320

[B17] Bugos J. A. Perlstein W. M. McCrae C. S. Brophy T. S. Bedenbaugh P. H. (2007). Individualized piano instruction enhances executive functioning and working memory in older adults. *Aging Ment. Health* 11 464–471. 10.1080/13607860601086504 17612811

[B18] Buhusi C. V. Meck W. H. (2005). What makes us tick? Functional and neural mechanisms of interval timing. *Nat. Rev. Neurosci.* 6 755–765. 10.1038/nrn1764 16163383

[B19] Camargo A. Azuaje F. Wang H. Zheng H. (2008). Permutation – based statistical tests for multiple hypotheses. *Source Code Biol. Med.* 3:15. 10.1186/1751-0473-3-15 18939983 PMC2611984

[B20] Chan T. M. V. Alain C. (2020). “Theories of cognitive aging: a look at potential benefits of music training on the aging brain,” in *Music and the Aging Brain*, eds Cuddy L. L. Belleville S. Moussard A. (Cambridge, MA: Academic Press), 195–220.

[B21] Chen Y. Lv C. Li X. Zhang J. Chen K. Liu Z. (2019). The positive impacts of early-life education on cognition, leisure activity, and brain structure in healthy aging. *Aging (Albany NY).* 11 4923–4942. 10.18632/aging.102088 31315089 PMC6682517

[B22] Cheng S. T. (2016). Cognitive reserve and the prevention of dementia: the role of physical and cognitive activity. *Curr. Psychiatry Rep.* 18:85. 10.1007/s11920-016-0721-2 27481112 PMC4969323

[B23] Colombo P. J. Habibi A. Alain C. (2020). Editorial: music training, neural plasticity, and executive function. *Front. Integr. Neurosci.* 14:41. 10.3389/fnint.2020.00041 32903753 PMC7438867

[B24] D’Agata F. Caroppo P. Boghi A. Coriasco M. Caglio M. Baudino B. (2011). Linking coordinative and executive dysfunctions to atrophy in spinocerebellar ataxia 2 patients. *Brain Struct. Funct.* 216 275–288. 10.1007/s00429-011-0310-4 21461742

[B25] Duan X. Liao W. Liang D. Qiu L. Gao Q. Liu C. (2012). Large-scale brain networks in board game experts: insights from a domain-related task and task-free resting state. *PLoS One* 7:e32532. 10.1371/journal.pone.0032532 22427852 PMC3299676

[B26] Dudoit S. Shaffer J. P. Boldrick J. C. (2003). Multiple hypothesis testing in microarray experiments. *Statist. Sci.* 18 71–103. 10.1214/ss/1056397487

[B27] Eickhoff S. B. Stephan K. E. Mohlberg H. Grefkes C. Fink G. R. Amunts K. (2005). A new SPM toolbox for combining probabilistic cytoarchitectonic maps and functional imaging data. *Neuroimage* 25 1325–1335. 10.1016/j.neuroimage.2004.12.034 15850749

[B28] Erickson K. I. Voss M. W. Prakash R. S. Basak C. Szabo A. Chaddock L. (2011). Exercise training increases size of hippocampus and improves memory. *Proc. Natl. Acad. Sci. U.S.A.* 108 3017–3022. 10.1073/pnas.1015950108 21282661 PMC3041121

[B29] Fauvel B. Groussard M. Mutlu J. Arenaza-Urquijo E. M. Eustache F. Desgranges B. (2014). Musical practice and cognitive aging: two cross-sectional studies point to phonemic fluency as a potential candidate for a use-dependent adaptation. *Front. Aging Neurosci.* 6:227. 10.3389/fnagi.2014.00227 25346684 PMC4191346

[B30] Firth J. Stubbs B. Vancampfort D. Schuch F. Lagopoulos J. Rosenbaum S. (2018). Effect of aerobic exercise on hippocampal volume in humans: a systematic review and meta-analysis. *Neuroimage* 166 230–238. 10.1016/j.neuroimage.2017.11.007 29113943

[B31] Gaab N. Gaser C. Schlaug G. (2006). Improvement-related functional plasticity following pitch memory training. *Neuroimage* 31 255–263. 10.1016/j.neuroimage.2005.11.046 16427320

[B32] Gaab N. Gaser C. Zaehle T. Jancke L. Schlaug G. (2003). Functional anatomy of pitch memory–an fMRI study with sparse temporal sampling. *Neuroimage* 19 1417–1426. 10.1016/s1053-8119(03)00224-6 12948699

[B33] Gao J. H. Parsons L. M. Bower J. M. Xiong J. Li J. Fox P. T. (1996). Cerebellum implicated in sensory acquisition and discrimination rather than motor control. *Science* 272 545–547. 10.1126/science.272.5261.545 8614803

[B34] Gaser C. Schlaug G. (2003). Brain structures differ between musicians and non-musicians. *J. Neurosci.* 23 9240–9245. 10.1523/JNEUROSCI.23-27-09240.2003 14534258 PMC6740845

[B35] Groussard M. La Joie R. Rauchs G. Landeau B. Chételat G. Viader F. (2010). When music and long-term memory interact: effects of musical expertise on functional and structural plasticity in the hippocampus. *PLoS One* 5:e13225. 10.1371/journal.pone.0013225 20957158 PMC2950159

[B36] Guo X. Yamashita M. Suzuki M. Ohsawa C. Asano K. Abe N. (2021). Musical instrument training program improves verbal memory and neural efficiency in novice older adults. *Hum. Brain Mapp.* 42 1359–1375. 10.1002/hbm.25298 33617124 PMC7927292

[B37] Hall C. B. Lipton R. B. Sliwinski M. Katz M. J. Derby C. A. Verghese J. (2009). Cognitive activities delay onset of memory decline in persons who develop dementia. *Neurology* 73 356–361. 10.1212/WNL.0b013e3181b04ae3 19652139 PMC2725932

[B38] Hanakawa T. Immisch I. Toma K. Dimyan M. A. Van Gelderen P. Hallett M. (2003). Functional properties of brain areas associated with motor execution and imagery. *J. Neurophysiol.* 89 989–1002. 10.1152/jn.00132.2002 12574475

[B39] Hanna-Pladdy B. Gajewski B. (2012). Recent and past musical activity predicts cognitive aging variability: direct comparison with general lifestyle activities. *Front. Hum. Neurosci.* 6:198. 10.3389/fnhum.2012.00198 22833722 PMC3400047

[B40] Hanna-Pladdy B. MacKay A. (2011). The relation between instrumental musical activity and cognitive aging. *Neuropsychology* 25 378–386. 10.1037/a0021895 21463047 PMC4354683

[B41] Harding I. H. Corben L. A. Delatycki M. B. Stagnitti M. R. Storey E. Egan G. F. (2017). Cerebral compensation during motor function in Friedreich ataxia: the IMAGE-FRDA study. *Mov. Disord.* 32 1221–1229. 10.1002/mds.27023 28556242

[B42] Hautzel H. Mottaghy F. M. Specht K. Müller H. W. Krause B. J. (2009). Evidence of a modality-dependent role of the cerebellum in working momory? An fMRI study comparing verbal and abstract n-back tasks. *Neuroimage* 47 2073–2082. 10.1016/j.neuroimage.2009.06.005 19524048

[B43] Heim S. Eickhoff S. B. Amunts K. (2008). Specialisation in Broca’s region for semantic, phonological, and syntactic fluency? *Neuroimage* 40 1362–1368. 10.1016/j.neuroimage.2008.01.009 18296070

[B44] Herholz S. C. Halpern A. R. Zatorre R. J. (2012). Neuronal correlates of perception, imagery, and memory for familiar tunes. *J. Cogn. Neurosci.* 24 1382–1397. 10.1162/jocn_a_00216 22360595

[B45] Hickok G. Buchsbaum B. Humphries C. Muftuler T. (2003). Auditory-motor interaction revealed by fMRI: speech, music, and working memory in area Spt. *J. Cogn. Neurosci.* 15 673–682. 10.1162/089892903322307393 12965041

[B46] Holm L. Karampela O. Ullén F. Madison G. (2017). Executive control and working memory are involved in sub-second repetitive motor timing. *Exp. Brain Res.* 235 787–798. 10.1007/s00221-016-4839-6 27885405 PMC5315705

[B47] Huang Q. Liu Y. Liao W. Yang S. Shen L. Tang T. (2020). Disruption of regional brain activity and functional connectivity in patients with asymptomatic vulnerable carotid plaque. *Neurosci. Lett.* 716:134634. 10.1016/j.neulet.2019.134634 31751668

[B48] Hutchinson S. Lee L. H. Gaab N. Schlaug G. (2003). Cerebellar volume of musicians. *Cereb. Cortex* 13 943–949. 10.1093/cercor/13.9.943 12902393

[B49] James C. E. Britz J. Vuilleumier P. Hauert C. A. Michel C. M. (2008). Early neuronal responses in right limbic structures mediate harmony incongruity processing in musical experts. *Neuroimage* 42 1597–1608. 10.1016/j.neuroimage.2008.06.025 18640279

[B50] Jäncke L. Schlaug G. Steinmetz H. (1997). Hand skill asymmetry in professional musicians. *Brain Cogn.* 34 424–432. 10.1006/brcg.1997.0922 9292190

[B51] Johnson R. B. (2011). Musical tempo stability in mental practice: a comparison of motor and non-motor imagery techniques. *Res. Stud. Music Edu.* 33 3–30. 10.1177/1321103X11400501

[B52] Karpati F. J. Giacosa C. Foster N. E. Penhune V. B. Hyde K. L. (2016). Sensorimotor integration is enhanced in dancers and musicians. *Exp. Brain Res*. 234 893–903. 10.1007/s00221-015-4524-1 26670906

[B53] Klein C. Liem F. Hänggi J. Elmer S. Jäncke L. (2016). The “silent” imprint of musical training. *Hum. Brain Mapp.* 37 536–546. 10.1002/hbm.23045 26538421 PMC6867483

[B54] Kraus N. Chandrasekaran B. (2010). Music training for the development of auditory skills. *Nat. Rev. Neurosci.* 11 599–605. 10.1038/nrn2882 20648064

[B55] Lerub K. D. Vines B. W. Shinde A. B. Schlaug G. (2021). Modulating short-term auditory memory with focal transcranial direct current stimulation applied to the supramarginal gyrus. *Neuroreport* 32 702–710. 10.1097/WNR.0000000000001647 33852539 PMC8085037

[B56] Lezak M. D. Howieson D. B. Bigler E. D. Tranel D. (2012). *Neuropsychological Assessment*, 5th Edn. New York, NY: Oxford University Press.

[B57] Lotze M. Scheler G. Tan H.-R. M. Braun C. Birbaumer N. (2003). The musician’s brain: functional imaging of amateurs and professionals during performance and imagery. *Neuroimage* 20 1817–1829. 10.1016/j.neuroimage.2003.07.018 14642491

[B58] Luo C. Guo Z. W. Lai Y. X. Liao W. Liu Q. Kendrick K. M. (2012). Musical training induces functional plasticity in perceptual and motor networks: insights from resting-state FMRI. *PLoS One* 7:e36568. 10.1371/journal.pone.0036568 22586478 PMC3346725

[B59] Mak M. K. Cheung V. Ma S. Lu Z. L. Wang D. Lou W. (2016). Increased cognitive control during execution of finger tap movement in people with Parkinson’s Disease. *J. Parkinsons Dis.* 6 639–650. 10.3233/JPD-160849 27372216

[B60] Meister I. G. Krings T. Foltys H. Boroojerdi B. Müller M. Töpper R. (2004). Playing piano in the mind–an fMRI study on music imagery and performance in pianists. *Brain Res. Cogn. Brain Res.* 19 219–228. 10.1016/j.cogbrainres.2003.12.005 15062860

[B61] Moreno S. Bialystok E. Barac R. Schellenberg E. G. Cepeda N. J. Chau T. (2011). Short-term music training enhances verbal intelligence and executive function. *Psychol. Sci.* 22 1425–1433. 10.1177/0956797611416999 21969312 PMC3449320

[B62] Mortimer J. A. Ding D. Borenstein A. R. DeCarli C. Guo Q. Wu Y. (2012). Changes in brain volume and cognition in a randomized trial of exercise and social interaction in a community-based sample of non-demented Chinese elders. *J. Alzheimers Dis.* 30 757–766. 10.3233/JAD-2012-120079 22451320 PMC3788823

[B63] Müllensiefen D. Gingras B. Musil J. Stewart L. (2014). The musicality of no-musicians: an index for assessing musical sophistication in the general population. *PLoS One* 9:e89642. 10.1371/journal.pone.0089642 24586929 PMC3935919

[B64] Notter M. P. Hanke M. Murray M. M. Geiser E. (2020). Encoding of auditory temporal gestalt in the human Brain. *Cereb. Cortex* 29 475–484. 10.1093/cercor/bhx328 29365070

[B65] Nyberg L. Lövdén M. Riklund K. Lindenberger U. Bäckman L. (2012). Memory aging and brain maintenance. *Trends Cogn. Sci.* 16 292–305. 10.1016/j.tics.2012.04.005 22542563

[B66] Olivito G. Lupo M. Iacobacci C. Clausi S. Romano S. Masciullo M. (2018). Structural cerebellar correlates of cognitive functions in spinocerebellar ataxia type 2. *J. Neurol.* 265 597–606. 10.1007/s00415-018-8738-6 29356974

[B67] Palmer C. Drake C. (1997). Monitoring and planning capacities in the acquisition of music performance skills. *Can. J. Exp. Psychol.* 51 369–384. 10.1037/1196-1961.51.4.369 9606950

[B68] Paquette S. Fujii S. Li H. C. Schlaug G. (2017). The cerebellum’s contribution to beat interval discrimination. *Neuroimage* 163 177–182. 10.1016/j.neuroimage.2017.09.017 28916178 PMC5972378

[B69] Parbery-Clark A. Strait D. L. Anderson S. Hittner E. Kraus N. (2011). Musical experience and the aging auditory system: implications for cognitive abilities and hearing speech in noise. *PLoS One* 6:e18082. 10.1371/journal.pone.0018082 21589653 PMC3092743

[B70] Park D. C. Lautenschlager G. Hedden T. Davidson N. S. Smith A. D. Smith P. K. (2002). Models of visuospatial and verbal memory across the adult life span. *Psychol. Aging* 17 299–320. 10.1037/0882-7974.17.2.299 12061414

[B71] Patel A. D. Iversen J. R. (2007). The linguistic benefits of musical abilities. *Trends Cogn. Sci.* 11 369–372. 17698406 10.1016/j.tics.2007.08.003

[B72] Patel A. D. Iversen J. R. (2014). The evolutionary neuroscience of musical beat perception: the action simulation for auditory prediction. *Front. Syst. Neurosci.* 8:57. 10.3389/fnsys.2014.00057 24860439 PMC4026735

[B73] Paulesu E. Frith C. D. Frackowiak R. S. (1993). The neural correlates of the verbal component of working memory. *Nature* 362 342–345. 10.1038/362342a0 8455719

[B74] Penhune V. B. Zatorre R. J. Evans A. C. (1998). Cerebellar contributions to motor timing: a PET study of auditory and visual rhythm reproduction. *J. Cogn. Neurosci.* 10 752–765. 10.1162/089892998563149 9831742

[B75] Ramanoël S. Hoyau E. Kauffmann L. Renard F. Pichat C. Boudiaf N. (2018). Gray matter volume and cognitive performance during normal aging. A voxel-based morphometry study. *Front. Aging Neurosci.* 10:235. 10.3389/fnagi.2018.00235 30123123 PMC6085481

[B76] Rauschecker J. P. (2011). An expanded role for the dorsal auditory pathway in sensorimotor control and integration. *Hear. Res.* 271 16–25. 10.1016/j.heares.2010.09.001 20850511 PMC3021714

[B77] Raz N. Ghisletta P. Rodrigue K. M. Kennedy K. M. Lindenberger U. (2010). Trajectories of brain aging in middle-aged and older adults: regional and individual differences. *Neuroimage* 51 501–511. 10.1016/j.neuroimage.2010.03.020 20298790 PMC2879584

[B78] Raz N. Gunning-Dixon F. Head D. Rodrigue K. M. Williamson A. Acker J. D. (2004). Aging, sexual dimorphism, and hemispheric asymmetry of the cerebral cortex: replicability of regional differences in volume. *Neurobiol. Aging* 25 377–396. 10.1016/S0197-4580(03)00118-0 15123343

[B79] Reitan R. M. Wolfson D. (1993). *Halstead-Reitan Neuropsychological Battery.* Tuscon, AZ: Neuropsychology Press.

[B80] Schmahmann J. D. (2004). Disorders of the cerebellum: ataxia, dysmetria of thought, and the cerebellar cognitive affective syndrome. *J. Neuropsychiatry Clin. Neurosci.* 16 367–378. 10.1176/jnp.16.3.367 15377747

[B81] Schmahmann J. D. (2019). The cerebellum and cognition. *Neurosci. Lett.* 688 62–75. 10.1016/j.neulet.2018.07.005 29997061

[B82] Schwartze M. Kotz S. A. (2013). A dual-pathway neural architecture for specific temporal prediction. *Neurosci. Biobehav. Rev.* 37 2587–2596. 10.1016/j.neubiorev.2013.08.005 23994272

[B83] Seidler R. D. Bernard J. A. Burutolu T. B. Fling B. W. Gordon M. T. Gwin J. T. (2010). Motor control and aging: links to age-related brain structural, functional, and biochemical effects. *Neurosci. Biobehav. Rev.* 34 721–733. 10.1016/j.neubiorev.2009.10.005 19850077 PMC2838968

[B84] Shao Z. Janse E. Visser K. Meyer A. S. (2014). What do verbal fluency tasks measure? Predictors of verbal fluency performance in older adults. *Front. Psychol.* 5:772. 10.3389/fpsyg.2014.00772 25101034 PMC4106453

[B85] Shen Y. Lin Y. Liu S. Frang L. Liu G. (2019). Sustained effect of music training on the enhancement of executive function in preschool children. *Front. Psychol.* 10:1910. 10.3389/fpsyg.2019.01910 31507486 PMC6714059

[B86] Stoodley C. J. (2012). The cerebellum and cognition: evidence from functional imaging studies. *Cerebellum* 11 352–365. 10.1007/s12311-011-0260-7 21373864

[B87] Stoodley C. J. Schmahmann J. D. (2009). Functional topography in the human cerebellum: a meta-analysis of neuroimaging studies. *Neuroimage* 44 489–501. 10.1016/j.neuroimage.2008.08.039 18835452

[B88] Stoodley C. J. Schmahmann J. D. (2018). Functional topography of the human cerebellum. *Handb. Clin. Neurol.* 154 59–70. 10.1016/B978-0-444-63956-1.00004-7 29903452

[B89] Strait D. L. Hornickel J. Kraus N. (2011). Subcortical processing of speech regularities underlies reading and music aptitude in children. *Behav. Brain Funct.* 7:44. 10.1186/1744-9081-7-44 22005291 PMC3233514

[B90] Strong J. V. Mast B. T. (2019). The cognitive functioning of older adult instrumental musicians and non-musicians. *Neuropsychol. Dev. Cogn. B. Aging Neuropsychol. Cogn.* 26 367–386. 10.1080/13825585.2018.1448356 29516767

[B91] Sugishita M. (2000). *Wechsler Memory Scale – Revised.* Tokyo: Nihon Bunka Kagakusha.

[B92] Trusheim W. H. (1991). Audiation and mental imagery: implications for artistic performance. *Q. J. Music Teach. Learn.* 2 138–147.

[B93] Tsai C. G. Chou T. L. Li C. W. (2018). Roles of posterior parietal and dorsal premotor cortices in relative pitch processing: comparing musical intervals to lexical tones. *Neuropsychologia* 119 118–127. 10.1016/j.neuropsychologia.2018.07.028 30056054

[B94] Tse N. Y. Chen Y. Irush M. Cordato N. J. Landin-Romero R. Hodges J. R. (2020). Cerebellar contributions to cognition in corticobasal syndrome and progressive supranuclear palsy. *Brain Commun.* 2:fcaa194. 10.1039/braincomms/fcaa194 33381758 PMC7753056

[B95] Vaquero L. Hartmann K. Ripollés P. Rojo N. Sierpowska J. François C. (2016). Structural neuroplasticity in expert pianists depends on the age of musical training onset. *Neuroimage* 126 106–119. 10.1016/j.neuroimage.2015.11.008 26584868

[B96] Verghese J. Lipton R. B. Katz M. J. Hall C. B. Derby C. A. Kuslansky G. (2003). Leisure activities and the risk of dementia in the elderly. *N. Engl. J. Med.* 348 2508–2516. 10.1056/NEJMoa022252 12815136

[B97] Wang Y. Fang J. L. Cui B. Liu J. Song P. Lang C. (2018). The functional and structural alterations of the striatum in chronic spontaneous urticaria. *Sci. Rep.* 8:1725. 10.1038/s41598-018-19962-2 29379058 PMC5789061

[B98] Wechsler D. (1997). *Manual for the Wechsler Adult Intelligence Scale III.* San Antonio, TX: Harcourt Assessment.

[B99] Weiss Y. Cweigenberg H. G. Booth J. R. (2018). Neural specialization of phonological and semantic processing in young children. *Hum. Brain Mapp.* 39 4334–4348. 10.1002/hbm.24274 29956400 PMC6261343

[B100] White-Schwoch T. Woodruff Carr K. Anderson S. Strait D. L. Kraus N. (2013). Older adults benefit from music training early in life: biological evidence for long-term training-driven plasticity. *J. Neurosci.* 33 17667–17674. 10.1523/JNEUROSCI.2560-13.2013 24198359 PMC3818545

[B101] Whitfield-Gabrieli S. Nieto-Castanon A. (2012). Conn: a functional connectivity toolbox for correlated and anticorrelated brain networks. *Brain Connect.* 2 125–141. 10.1089/brain.2012.0073 22642651

[B102] Wieser H. G. Mazzola G. (1986). Musical consonances and dissonances: are they distinguished independently by the right and left hippocampi? *Neuropsychologia* 24 805–812. 10.1016/0028-3932(86)90079-5 3808288

[B103] Wollman I. Penhune V. Segado M. Carpentier T. Zatorre R. J. (2018). Neural network retuning and neural predictors of learning success associated with cello training. *Proc. Natl. Acad. Sci. U.S.A.* 115 E6056–E6064. 10.1073/pnas.1721414115 29891670 PMC6042146

[B104] Wong P. C. Skoe E. Russo N. M. Dees T. Kraus N. (2007). Musical experience shapes human brainstem encoding of linguistic pitch patterns. *Nat. Neurosci.* 10 420–422. 10.1038/nn1872 17351633 PMC4508274

[B105] Wu X. Zhang R. Li X. Feng T. Yan N. (2021). The moderating role of sensory processing sensitivity in the link between stress and depression: a VBM study. *Neuropsychologia* 150:107704. 10.1016/j.neuropsychologia.2020.107704 33276034

[B106] Zatorre R. J. Chen J. L. Penhune V. B. (2007). When the brain plays music: auditory-motor interactions in music perception and production. *Nat. Rev. Neurosci.* 8 547–558. 10.1038/nrn2152 17585307

[B107] Zendel B. R. Alain C. (2014). Enhanced attention-dependent activity in the auditory cortex of older musicians. *Neurobiol. Aging* 35 55–63. 10.1016/j.neurobiolaging.2013.06.022 23910654

