# Supplementary material for: Neural Advantages of Older Musicians Involve the Cerebellum: Implications for Healthy Aging Through Lifelong Musical Instrument Training
Source: Front Hum Neurosci. 2022 Jan 5;15:784026. doi: 10.3389/fnhum.2021.784026 (PMC8766763; doi:10.3389/fnhum.2021.784026)
Supplement: Supplementary file 1 [file Data_Sheet_1.docx]

Supplementary Material

# Supplementary Figures and Tables

## Supplementary Figures

**Supplementary Figure 1.** Structural changes in the hippocampus. (A) Musicians had a larger GMV in the right hippocampus compared to non-musicians when using an uncorrected statistical threshold. (B) For the hippocampal ROI in the right hemisphere, the 60 older participants, including musicians and non-musicians, showed negative correlations between their GMV and age. * *P* < 0.05, GMV, gray matter volume; ROI, region of interest; L, left; R, right; a.u., arbitrary units.

## Supplementary Tables

Supplementary Table 1. Lifestyles for each group

| Lifestyle type |  |
| --- | --- |
| Musicians |  |
| Exercise | Walking, aerobics, muscle training, table tennis, golfing, bowling, gymnastics, cycling, skiing, stretching, billiards, swimming, diving, tennis, yoga, qigong, dancing |
| Cognitive activity | Embroidery, tea ceremony, flower arrangement, photography, sake brewing, fishing, handcraft, calligraphy, motoring, personal computer activities, dressing (kimono), board games, English conversation, knitting, painting, reading |
| Non-musicians |  |
| Exercise | Walking, golfing, Chinese shadow boxing, muscle training, stretching, tennis, gymnastics, dancing, volleyball, qigong |
| Cognitive activity | Painting, handcraft, board games, movie appreciation, video gaming, English conversation, cooking classes, tea ceremony, attending history workshops, flower arrangement, music appreciation, drama, engraving, reading, painting appreciation, Japanese dressmaking, personal computer activities, sightseeing, calligraphy |

Supplementary Table 2. Brain areas with significantly larger gray matter volume in musicians compared to non-musicians (cluster-level *P* < 0.05, FWE corrected)

|  | MNI coordinates | | |  |  |
| --- | --- | --- | --- | --- | --- |
| Location | x | y | z | *Z*-value | Cluster size |
| Left cerebellum | −29 | −63 | −36 | 4.34 | 966 |
| Right cerebellum | 27 | −65 | −36 | 4.25 | 954 |

FWE, family-wise error; MNI, Montreal Neurological Institute.

Supplementary Table 3. Task-related FC (seed in the left cerebellum) with significantly higher values in musicians compared to non-musicians (cluster-level *P* < 0.05, FWE corrected)

|  | MNI coordinates | | |  |  |
| --- | --- | --- | --- | --- | --- |
| Location | x | y | z | *Z*-value | Cluster size |
| Right hippocampus | 24 | −10 | −16 | 5.07 | 178 |

FC, functional connectivity; FWE, family-wise error; MNI, Montreal Neurological Institute.

Supplementary Table 4. Peak coordinates of group differences (musicians > non-musicians) for fMRI activation during the MWM task (1-back vs. rest; cluster-level *P* < 0.05, FWE corrected)

|  | MNI coordinates | | |  |  |
| --- | --- | --- | --- | --- | --- |
| Location | x | y | z | *Z*-value | Cluster size |
| Left supramarginal gyrus | −52 | −34 | 24 | 4.22 | 821 |

fMRI, functional magnetic resonance imaging; MWM, melodic working memory; FWE, family-wise error; MNI, Montreal Neurological Institute.

Supplementary Table 5. Brain areas with significantly larger gray matter volume in musicians compared to non-musicians (voxel-level *P* < 0.001, uncorrected)

|  | MNI coordinates | | |  |  |
| --- | --- | --- | --- | --- | --- |
| Location | x | y | z | *Z*-value | Cluster size |
| Right hippocampus | 26 | −36 | −8 | 4.08 | 246 |

MNI, Montreal Neurological Institute.
